# Mental Health Among School-Going Adolescents in Greater London: A Cross-Sectional Study

**DOI:** 10.3389/fpsyt.2021.592624

**Published:** 2021-03-19

**Authors:** Asmaa Al-Zawaadi, Iman Hesso, Reem Kayyali

**Affiliations:** School of Life Sciences, Pharmacy and Chemistry, Kingston University London, Kingston upon Thames, United Kingdom

**Keywords:** mental health, adolescents', risk factors, causes, bullying, discrimination

## Abstract

**Objectives:** Mental health problems are among the leading causes of health-related disability among children and adolescents worldwide. However, there is still a global challenge in terms of gathering consistent epidemiological information about the issue. The present study was designed to describe various mental health issues and factors associated with negative feelings among adolescents in Greater London.

**Methods:** This is a cross sectional study, using a self-administered questionnaire (Cronbach's alpha = 0.742). A convenience sampling strategy was used to recruit participants who were school/college-attending adolescents, aged 11–19. A minimum sample size of 199 was required (95% confidence interval, 5% margin of error, and 15.3% population proportion). The study was conducted between February and April 2016 in Greater London. Descriptive statistics and inferential statistics including chi square, Spearman correlation, and binary logistic regression were used to identify the key findings. Data analysis was performed using SPSS v21.

**Results:** A total of 526 out of 1,920 surveys were collected across 18 secondary schools and two colleges, giving a response rate of 27.4%. More than half of the adolescents reported to be either neutral (41.4%), sad (7.8%), or very sad (2.8%), whereas 48% reported to be either happy (35%) or very happy (13%). Difficulties in relationships and hectic schedules were among the most stressful situations affecting adolescents' mental health. Discrimination was identified as the main predicting factor with five-fold increase in odds of having negative mental health symptomatology. Other significant risk factors identified were age, gender, smoking, and health comorbidities.

**Conclusion:** Discrimination was identified as the most predictive factor influencing negative symptomatology among the study cohort. The study had several limitations, most notably the use of a non-validated surrogate measure for mental health, in addition to the exclusion of adolescents aged 10–11 years, school/college dropouts and non-school-going adolescents. A similar study on a national scale is highly recommended to determine the real magnitude of the problem, which would be the starting point toward proper tackling of mental health issues and associated complexities among the adolescent population across England.

## Introduction

Mental health problems are among the leading causes of health-related disability among children and adolescents worldwide (1). The prevalence of mental health disorders has been reported to increase in adolescence. Worldwide, estimates indicate that up to 20% of children and adolescents have mental health disorders, which accounts for a large portion of the global burden of the disease (1, 2). In Europe, mental health disorders have an estimated prevalence of 8–23% of the child and adolescent population (3). Adolescence is a key period in life for establishing the foundation for health and well-being in adulthood (4). Existing evidence in the literature suggests that a substantial proportion of mental health problems in adults originate in mid-to-late adolescence and contribute to the existing burden of the disease among young people and in later life (1, 5); three quarters of mental health problems start before the early 20s (4, 6).

There are 7.4 million young people aged 10–19, representing 11% of the total population of the United Kingdom (UK) in 2017, with 21.5% estimated to be from an ethnic minority (6). According to 2017 estimates, young people aged 10–19 live in 4.8 million households, mainly with married parents (62%), lone parents (23%), or cohabitating parents (9%), and 1.9 million are living in the most deprived areas (6). In the UK, the British Child and Adolescent Mental Health Surveys in 1999 and 2004 reported that 1 in 10 children and young people under the age of 16 had a diagnosed mental disorder. Among the 11–16-year-old age group, 13% of boys and 10% of girls had a mental health issue; most commonly conduct disorders, hyperkinetic, emotional disorder, and autism spectrum disorder (ASD) (4, 7). The most common problems for boys are conduct disorders, whereas the most common problems for girls are emotional disorders (4). The more recent estimates in England showed that one in seven adolescents (15.3%) aged 11–19 met the criteria for having a mental health disorder at the time of the 2017 Mental Health of Children and Young People Survey (8, 9). A very recent follow-up survey was conducted in July 2020 during the coronavirus-2019 (COVID-19) pandemic which showed that 17.6% of adolescents (one in six) aged 11–16 years were identified with a probable mental health disorder, an increase from 12.6% of adolescents (one in eight) in 2017 (9). According to the more recent statistics, emotional disorders such as anxiety and depression were found to be the most common mental disorders experienced by young people (8, 9). The economic burden associated with mental health disorders during childhood and adolescence is substantially high. In Europe, the economic consequences of child and adolescent mental health disorders are significant in terms of health services, social services, education system, criminal justice system, voluntary services, and productivity costs. The mean overall societal costs were estimated to range from €7,376 to €64,703 per child/adolescent in Europe annually (3). In the UK, mental health disorders were estimated to result in increased costs of between £11,030 and £59,130 annually per child including direct costs to families and costs related to education, social services, and youth justice (7).

Several factors have been identified to influence mental health among adolescents, including bullying (10–12), discrimination (13–18), low socioeconomic status (19, 20), low physical activity, high screen time (ST) (12, 21–27), smoking, and alcohol drinking (28–30). Poor family functioning has also been reported in the literature to have a robust link to poor adolescent mental health (31).

Mental health problems have important implications on various aspects of adolescents' lives including their ability to engage with education, engage in constructive family relationships, and make and keep friends in addition to developing self-dependence. Hence, detection, treatment, and support are all fundamental parts of the services to be provided to this young population (4). Despite the increased recognition of the prevailing burden and negative impact of mental health disorders among children and adolescents (3, 5), a global challenge still exists in terms of the capacity to gather consistent epidemiological information about the issue. The latter is important in order to provide a context for understanding the magnitude of this clinical problem, which in return would allow the identification of gaps in the services to support child/adolescent mental health, the quantification of child and adolescent mental disorders, the economic costs of impairment, or the lost potential for the individual or society (2). Therefore, this study aimed to describe the various mental health issues and factors contributing to them among adolescents in Greater London.

## Methods

### Research Design

This is a quantitative cross-sectional survey study among adolescents in schools and colleges. The study was conducted across four boroughs in London: Kingston upon Thames, Richmond upon Thanes, Croydon, and Wandsworth.

### Participants and Recruitment

A convenience sampling strategy was used when approaching schools/colleges to recruit participants. The total number of adolescents aged 11–19 years in the four boroughs was 104,000. The maximum prevalence of mental health disorders as determined by the 2017 statistics for young people aged 11–19 in England is 15.3% (8). The target sample size needed to achieve the study objectives with a sufficient statistical power of 80% was calculated using the Raosoft sample size calculator. Hence, the required sample size was 199 participants using a margin of error of 5%, a confidence interval of 95%, and a 15.3% population proportion.

Data collection took place between February and April 2016. A letter was designed for head teachers and students' well-being counselor to introduce the project, explain the purpose of the survey, and get the schools' permission to participate in the study.

Surveys were either sent to the school and then collected once completed or conducted directly by one of the research teams inside the school/college. Participants were informed about the study via an information sheet, which was attached with every single questionnaire. The information sheet provided details about the research aims, the questionnaire content, confidentiality, choice of participation, and withdrawal, as well as the contact details of the research team. Completion of the questionnaire indicated implied consent on the part of the student. Only fully completed questionnaires were included in the final analysis.

### Data Collection Tool

Data collection was performed using a paper-based questionnaire, consisting of 31 questions in total, covering four sections. The questionnaire was developed by the authors to address the study aims. The questionnaire consisted mostly of closed-ended questions including tick-box, pictorial, multiple-response, and contingency questions. The themes of the tool were adopted from the Renfrewshire community health partnership “Health and well-being survey of young people in Renfrewshire 2013” which aimed at establishing baseline information on the health and well-being of pupils aged 13–18 years across secondary schools in Renfrewshire (32). The first section consisted of seven questions (questions 1–7) covering demographics. The second section included seven questions (questions 8–14) investigating the participants' lifestyle. The third section comprised 11 questions (questions 15–25), which aimed at evaluating adolescents' mental health. In this section, adolescents were asked to rate their general feelings via a pictorial question with emoji facial expressions (question no. 15) on a scale from 1 to 5 with one indicating very happy to five indicating very sad. In addition, the third section included five multiple-response questions (questions 19, 22, 23, 24, and 25) comprising 15 statements signifying negative mental health feelings/symptomatology associated with common mental health disorders which are anxiety and depression, in order to identify the frequency of these feelings among adolescents in the last 6 months. The questions (19, 22, 23, 24, and 25) captured core symptomatologies/feelings associated with the two common mental health disorders: anxiety and depression. Across the questions, some symptomatologies were asked more than once; hence, the symptomatologies captured within these questions and the weighting of each feeling/symptomatology were as follows: sadness (weight 2-mentioned twice), feeling nervous, anxious or on edge (weight 2), feeling restless (weight 1), pervasive low mood (weight 2), loss of pleasure in activities (weight 1), feeling guilty or worthless (weight 1), marked change in appetite, weight or sleep (weight 3), tiredness and poor concentration (weight 2), and thoughts of dying or suicide (weight 1).

The rest of the questions in section three explored participants' experiences about aspects such as challenging situations in addition to discrimination and bullying. The last section included six questions (questions 26–31) focusing on adolescents' usage of social media and ST. The questionnaire took an average of 5–8 min to complete.

The questionnaire items were evaluated for internal reliability using Cronbach's α. Cronbach's alpha coefficient was 0.742, which is within the recommended range of 0.7–0.9 (33, 34).

### Pilot Study

After attaining ethical approval, a pilot study was conducted among five adolescents in a college in Kingston for assessing the face and content validity of the questionnaire. The outcome of the pilot indicated the need for some minor amendments of some questions. To avoid any type of bias, the questionnaires filled in the pilot study were excluded from the final analysis.

### Data Analysis

All responses were coded and entered into SPSS for statistical analysis. Descriptive statistics were carried out to describe participants' characteristics and data regarding the basic features of the study. Proportions/percentages were used to describe categorical variables. Comparisons of proportions between groups were performed using Chi-square tests. Adolescents' feeling scorings were calculated based on the sum of responses obtained from the symptomatology questions (questions 15, 19, 22, 23, 24, and 25) included in section three. Each response reflecting a negative symptomatology feeling indicated a score of one with a maximum score of 16 that can be obtained via the summation of the responses for the five questions (Table 1).

**Table 1:** Questions addressing negative symptomatology in the study questionnaire.

**Question**	**Negative symptomatology feeling phrases**
Q15	1. General feeling (Neutral/Sad/Very sad)
Q19	2. I was frequently feeling up and down
	3. I lost or gained weight without trying to, or my appetite have changed
	4. I felt frequently sad, like I couldn't go to school
	5. I felt slowed down compared to my usual pace
	6. I stopped having fun doing things that I used to enjoy
Q22	7. Panic attacks (Yes)
Q23	8. Low in mood (Yes)
Q24	9. I was feeling anxious, worried or scared about a lot of things in my life
	10. I felt that my worry was out of control
	11. I felt restless, agitated and tense
	12. I had troubles sleeping
	13. I could not fall or stay asleep
Q25	14. I felt exhausted
	15. I felt worthless or guilty
	16. I keep thinking about death

*Each response indicates a score of one. A maximum score of 16 can be obtained based on the summation of responses from all the questions*.

The correlation between general feeling status among participants and feeling scorings was determined using Spearman's rank correlation coefficient, with data about feeling scorings being categorized/dichotomized based on a cutoff point of 50% to establish dominance of negative feelings and identify causing factors. Hence, a feeling score of ≥50% indicates dominance of negative mental health, whereas a score of <50% indicates less dominance of negative mental health. Furthermore, ST was classified based on the recommended 2-h limit per day for adolescents (24, 25).

Furthermore, a multiple binary logistic regression was conducted to determine risk factors influencing mental health based on categorization of feeling scorings, with results being expressed as odds ratios (ORs) with 95% confidence limits. The set of possible predictors considered was age, gender, ethnicity, birth, weekly income, physical activity, ST, presence of health conditions, smoking, drinking, living with parents, bullying, and discrimination. *P-*values below 0.05 indicated statistical significance for all analyses.

### Ethical Approval

Ethical approval to conduct this study was granted from the Delegated Research Ethics Committee at the academic institution of the corresponding author (Ref: 1213/045).

## Results

### Response Rate, Demographics, and Lifestyle

Forty-four primary and secondary schools and colleges were approached. Twenty secondary schools and colleges agreed to participate, whereas all primary schools in the study areas denied participation given the sensitivity of the topic. Therefore, the age range of participants was 11–19, given that the starting age of students at secondary schools in England is 11 years. In total, 1920 surveys were distributed across the schools and colleges and 526 surveys were collected, giving a response rate of 27.4%. Participants' demographics are summarized in Table 2. Interestingly, only 8% (*n* = 41/526) of the surveyed adolescents follow the recommendations indicated by the National Institute for Health and Care Excellence (NICE) guidelines for children and young people which is playing sports 7 days a week (35). On the other hand, 17% of the adolescents reported to be currently smoking and nearly half (49%) reported to have health problems (Table 2).

**Table 2:** Demographics of study participants.

**Characteristics**	**Number**	**Percentage**
**Gender**		
Male	251	48
Female	275	52
**Age**		
11–15 years	172	33
16–19 years	354	67
**Birthplace**		
UK	376	71.5
Outside UK	150	28.5
**Ethnicity**		
White	257	49
Indian	15	3
Pakistani	29	5.5
Bangladeshi	8	1.5
African Black	62	12
Caribbean Black	53	10
Other black	13	2.5
Chinese	7	1
Other Asian	50	9.5
Persian	10	2
Arab	22	4
**Smoking**		
Yes	89	17
No	437	83
**Drinking**		
Yes	222	42
No	304	58
**Health problem**		
Yes	257	49
No	269	51
**Type of health problem**		
Physical disability	102	19
Diabetes	3	1
ADHD	16	3
Dyslexia	76	14
Eczema	54	10
Epilepsy	4	1
Autism/ASD	9	2
Painful joints	51	10
Learning difficulties	69	13
Other	257	49
None	269	51
**Physical activity (days/week)**		
<7 days a week	485	92
7 days a week	41	8
**Weekly income**		
Yes	293	56
No	233	44

*Total N = 526*.

Participants were asked about the amount of time they spend on laptop/phone per day. Nearly two third of the study cohort (68.1%, *n* = 358/526) reported to have a ST ≥2 h/days.

### General Feelings Status and Feeling Scorings

Students were asked to rate their general feelings via a pictorial question with emoji facial expressions. The majority of respondents indicated feeling neutral (41.4%, *n* = 218/526), followed by 35% (*n* = 184/526) feeling happy, 13% (*n* = 68/526) feeling very happy, with least percentages for being sad (7.8%, *n* = 41/526), or very sad (2.8%, *n* = 15/526). The participants were clustered based on feeling scorings, with scores of ≥ 50% (i.e., score ≥8/16) indicating dominance of negative symptomatology. Three quarters of the participants (74%, *n* = 387/526) had a feeling scoring of <50% indicating less dominance of negative symptomatology, whereas a quarter (26%, *n* = 139/526) had a feeling scoring of ≥ 50% indicating a negative mental feeling. A significant positive association was found between general feeling status in terms of expressing sad feelings and feeling scoring of ≥50% (*rs* = 0.374, *p* < 0.0001).

**Figure 1:**
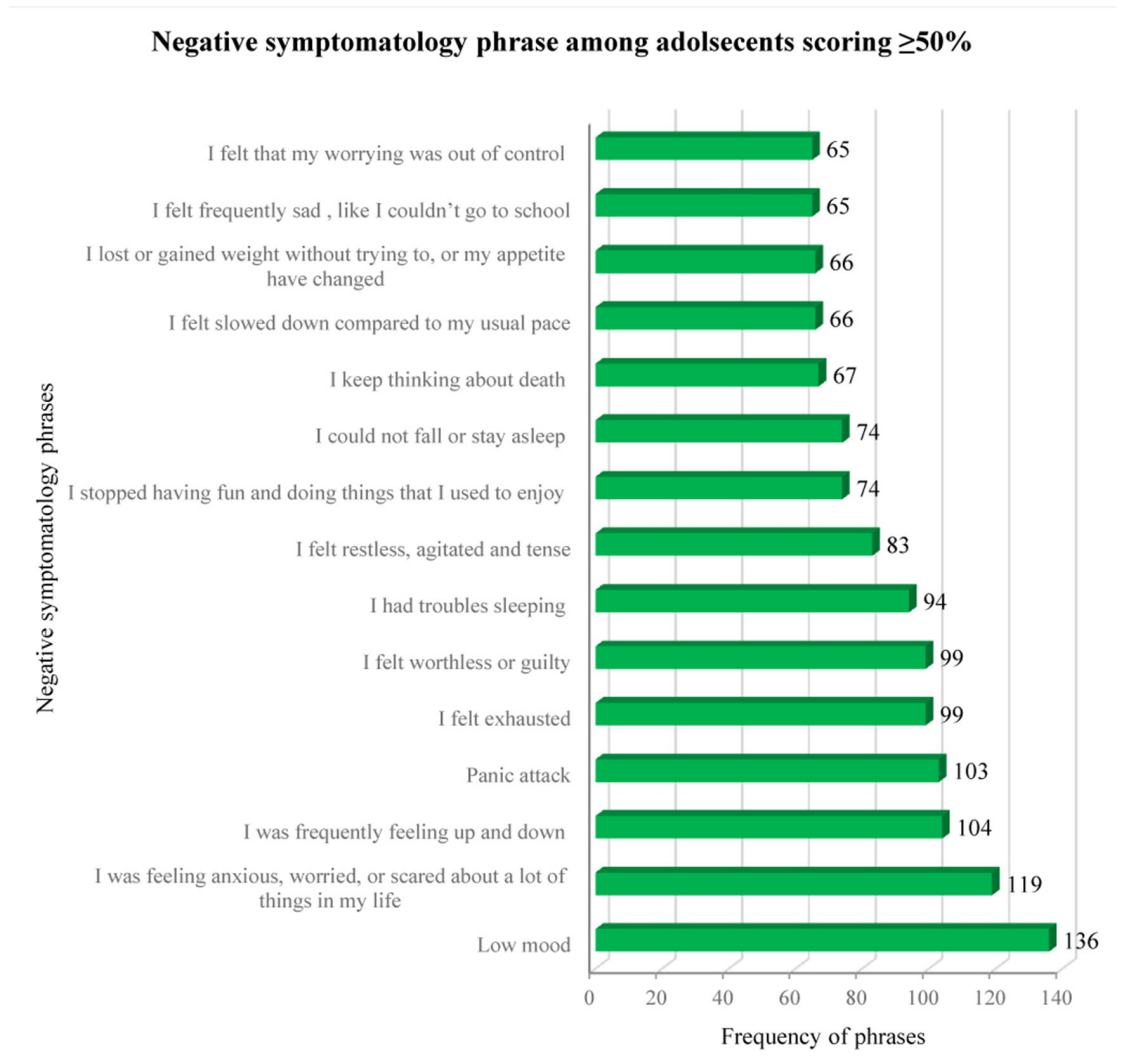
Frequency of negative symptomatology phrases among adolescents with feeling scorings ≥50%.

Further analysis was conducted among adolescents scoring ≥50% (*n* = 139) to identify the frequency of negative symptomatology phrases. The vast majority (97.8%, *n* = 136/139) reported being in low mood in the past 6 months. This was followed by feeling anxious, worried, or scared about a lot of things in life (85.6%, *n* = 119/139), frequently feeling up and down (74.8%, *n* = 104/139), and having panic attacks in the last 6 months (74.1%, *n* = 103/139). Equal responses were obtained for feeling exhausted and feeling worthless or guilty (71.2%, *n* = 99/139). Interestingly, more than two-thirds (67.6%, *n* = 94/139) reported to have trouble in sleeping and more than half (53.2%, *n* = 74/139) reported difficulties in falling or staying asleep. Feeling restless, agitated, and tense was reported by 59.7% (*n* = 83/139), and more than half (53.2%, *n* = 74/139) mentioned to stop having fun and doing enjoyable things. Another important negative symptomatology reported by nearly half of the adolescents (48.2%, *n* = 67/139) was related to the constant thinking about death (Figure 1).

### Main Causes of Mental Health Problems

Participants were asked about the most challenging and stressful situations that could negatively affect their mental health using a multiple-choice question, which provided 962 responses in total. Relationship difficulties was the main struggle for the young population with 20.5% (*n* = 197/962) responses, followed by hectic schedules (15.9%, *n* = 153/962). Sudden change, discrimination, and financial hardships were reported by 14.6% (*n* = 140/962), 12.4% (*n* = 119/962), and 11.9 % (*n* = 114/692), respectively. Feelings of isolation and serious challenges were equally identified as causes of stress with 10.3% (*n* = 99/962) and 10.2% (*n* = 98/962) of responses, respectively. Unsafe neighborhood was the least reported with 4.4% (*n* = 42/962).

Experiences and causes of discrimination and bullying were further elicited from the participants via multiple-response questions. A total of 60% (*n* = 316/526) of the adolescents reported to have experienced discrimination in the past 6 months. Body size, appearance, ethnicity, and skin color were the most reported causes of discrimination, with 18.8% (*n* = 157/833), 13% (*n* = 108/833), 12% (*n* = 100/833), and 11% (*n* = 92/833) of responses, respectively. This was followed by accent (9.6%, *n* = 80/833), gender (9.5%, *n* = 79/833), nationality (7.9%, *n* = 66/833), age (7.3%, *n* = 61/833), and language (5.3%, *n* = 44/833). Discrimination based on sexual orientation and disability were the least reported with 2.6% (*n* = 22/833) and 2.2% (*n* = 18/833) of responses, respectively. As for bullying, the majority of participants (78.3%, *n* = 412/526) did not report any form of bullying in the past 6 months. Among those who were bullied (21.7%, *n* = 114/526), this occurred mostly at school (65.8%, *n* = 75/114), followed by other places (22.8%, *n* = 26/114), thereafter at home (11.4%, *n* = 13/114).

### Factors Influencing Mental Health

#### Chi-Square Test of Association of Sociodemographic, Discrimination, and Bullying With Dominance of Negative Mental Feelings

The chi-square test revealed significant associations between dominance of negative mental feelings and each of age, gender, smoking, drinking, bullying, physical activity, discrimination, health conditions, and family instability (not living with both parents) (Table 3). Around a third of the female adolescents (33.5%) and those who drink (36.5%) reported dominance of negative mental feelings. While only 10.5% of young people aged below 16 reported feelings scoring ≥50%, Table 3 shows that this percentage was more than triple (34%) in those aged above 16. Additionally, 22% of non-smoking adolescents had feelings scoring ≥50%; however, the percentage was more than double (48.3%) in those who were smoking. Interestingly, dominance of negative mental health was reported by 39.2% of adolescents who experienced discrimination vs. only 7.1% who did not report any form of discrimination. As for bullying, 22% of adolescents who did not experience bullying in the last 6 months reported dominance of negative mental feelings, vs. 40.4% of those who did (Table 3). Nearly a third (32.1%) of adolescents not living with both parents had feelings scoring ≥50%, vs. 23.4% of those living with their parents.

**Table 3:** Association of sociodemographic factors, bullying, and discrimination with mental health (dominance of negative mental feelings).

**Participants' characteristics**		**Feeling scoring ≥50%, *n* (%)**	**Chi square (*X*^**2**^)**	***P*-value**
Gender	Male	47 (18.3%)	***X***^**2**^ (1) = 14.64	<0.0001
	Female	92 (33.5%)		
Age	<16	18 (10.5%)	***X***^**2**^ (1) = 33.48	<0.0001
	≥ 16	121 (34%)		
Birth place	In the UK	100 (26.6%)	***X***^**2**^ (1) = 0.02	0.889
	Outside the UK	39 (26%)		
Smoking	Yes	43 (48.3%)	***X***^**2**^ (1) = 26.39	<0.0001
	No	96 (22%)		
Drinking	Yes	81(36.5%)	***X***^**2**^ (1) = 19.99	<0.0001
	No	58 (19.1%)		
Weekly income	Yes	77 (26.3%)	***X***^**2**^ (1) =0.007	0.932
	No	62 (26.6%)		
Ethnicity	Black	26 (26.2%)	***X***^**2**^ (1) = 0.003	0.957
	Non-Black	112 (26.5%)		
Ethnicity	White	71 (26.2%)	***X***^**2**^ (1) = 0.015	0.903
	Non-white	68 (26.7%)		
Physical activity	Below recommended guidelines	134 (27.6%)	***X***^**2**^ (1) = 4.63	0.031
	Above recommended guidelines	5 (12.2%)		
Health conditions	Yes	89 (34.6%)	***X***^**2**^ (1) = 17.39	<0.0001
	No	50 (18.6%)		
Living with	Both parents	80 (23.4%)	***X***^**2**^ (1) =4.62	0.031
	Others	59 (32.1%)		
Bullying	Yes	46 (40.4%)	***X***^**2**^ (1) = 14.51	<0.0001
	No	93 (22.6%)		
Discrimination	Yes	124 (39.2%)	***X***^**2**^ (1) =66.85	<0.0001
	No	15 (7.1%)		
Screen time	≤ 2 h	29 (17.3%)	***X***^**2**^ (1) = 10.66	0.001
	>2 h	110 (30.7%)		

Ethnicity, birthplace, and income were not associated with dominance of negative mental feelings.

#### Multivariate Association of Sociodemographic, Discrimination, and Bullying With Dominance of Negative Mental Feelings

Certain factors were found to influence mental health in the study population, as indicated by the dominance of negative feeling (feeling scoring ≥ 50%). Gender was found to be a significant influencing factor, with female participants having ~2 odds more than males at scoring ≥ 50% (*p* = 0.01). Participants aged ≥16 were found to be 2.46 times more likely to have feeling scoring ≥ 50%, indicating age to be another significant factor (*p* = 0.01) (Table 4).

**Table 4:** Binary logistic analysis for risk factors affecting mental health in adolescents.

	**Dominance of negative mental health feelings/symptomatology associated with common mental health disorder which are anxiety and depression (feeling scoring** **≥50%)**		
**Characteristics**	**OR**	**95% CI**	***P*-value**
Gender (female)	1.85	1.16–2.94	0.01
Age (≥16 years)	2.46	1.23–4.92	0.01
Birthplace place in the UK	1.09	0.64–1.83	0.746
Smoking	1.82	1.04–3.21	0.036
Drinking	1.07	0.64–1.8	0.780
Presence of weekly income	0.73	0.45–1.17	0.196
Physical activity (below recommended guidelines)	1.57	0.54–4.56	0.407
Ethnicity (black)	0.68	0.35–1.31	0.253
Ethnicity (white)	1.19	0.68–2.05	0.533
Presence of health conditions	1.66	1.04–2.65	0.033
Living with both parents	0.90	0.57–1.44	0.685
Bullying	1.44	0.86–2.39	0.157
Discrimination	5.18	2.80–9.52	<0.0001
Screen time >2 h	1.34	0.77–2.31	0.293

Remarkably, participants who experienced discrimination in the last 6 months were 5.18 times more likely to have feeling scoring ≥ 50% (*p* < 0.0001). The odds of having feeling scoring ≥ 50% was 1.82 times higher in adolescents who were smoking (*p* = 0.036). Adolescents suffering from a health condition had an odds ratio of 1.66 for scoring ≥ 50% (*p* = 0.033). High ST (≥2 h/days), bullying, and doing less physical activity were found to increase the likelihood of scoring ≥50% by 1.34, 1.44, and 1.57 times, respectively, yet the effect of these factors was not significant (Table 4). Additionally, factors such as ethnicity, birthplace, income, and drinking were not found to have any significant effect on adolescents having negative mental health symptomatology (Table 4).

### Use of Social Media and Sources of Support Among Adolescents

In general, the most commonly used social media sites among adolescents were YouTube with 22.4% of responses (*n* = 357/1,592), followed by Instagram (20.2%, *n* = 322/1,592), WhatsApp (17.5%, *n* = 279/1,592), and Snapchat (16.8%, *n* = 268/1,592). Facebook and Twitter were the least used social media sites.

**Figure 2:**
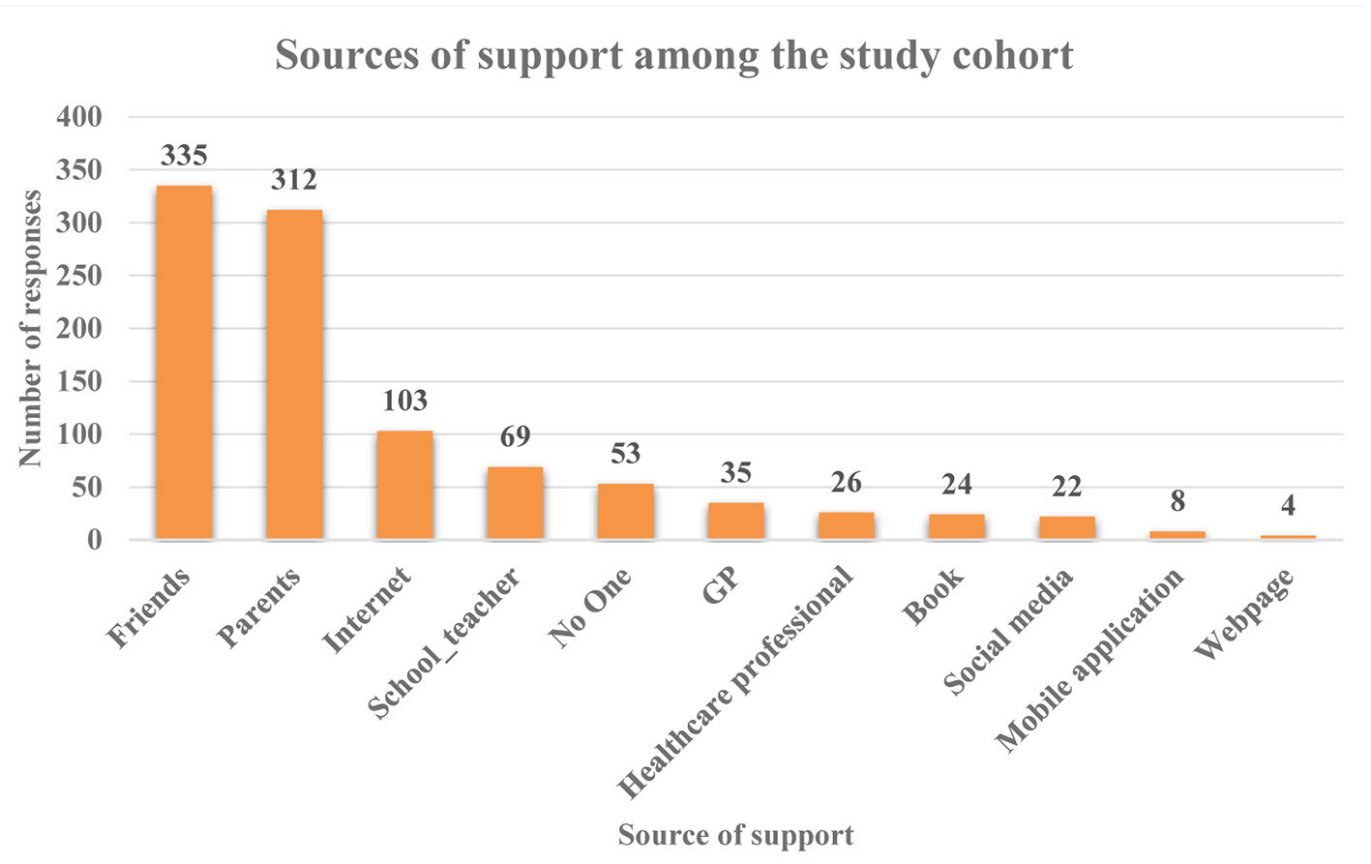
Sources of support among the study cohort. Number of responses, *n* = 991.

Identification of main sources of support was acquired from participants by means of a multiple-response question, which yielded a total of 991 responses. Friends were identified to be the main source of support among the surveyed adolescents with 33.8% of responses (*n* = 335/991) followed by parents (31.5%, *n* = 312/991). The third main form of support was the internet (10.4%, *n* = 103/991), followed by schoolteachers (7%, *n* = 69/991). The option “No one” attained 53 responses, constituting 5.3% of responses (Figure 2).

**Figure 3:**
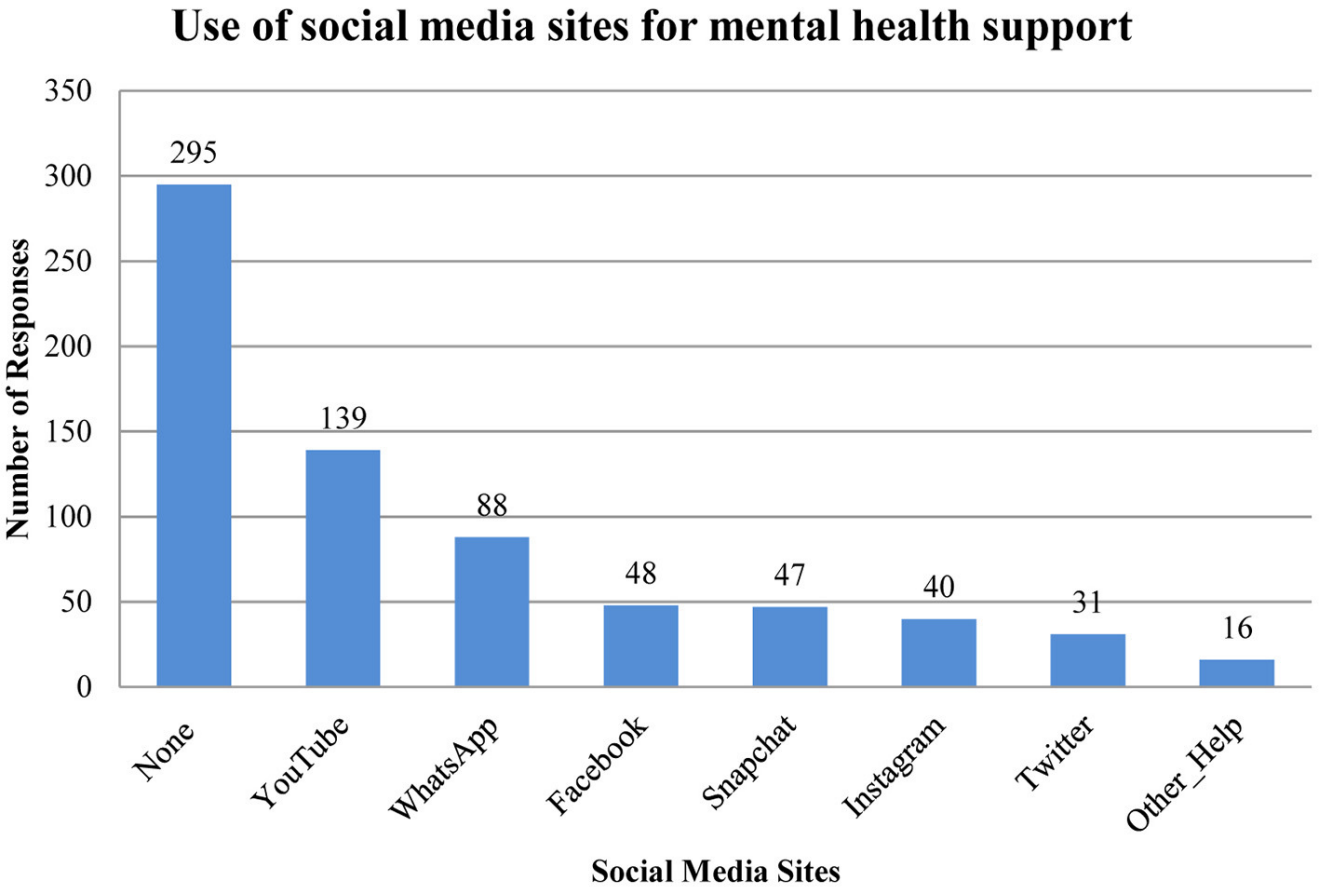
Use of social media sites among the study cohort for mental health support. Number of responses, *n* = 704.

Social media was not reported as a main source of support (Figure 2). However, when adolescents were specifically asked about the use of social media in relation to mental health, one-third (34%, *n* = 179/526) reported to share mental health problems on social media sites, vs. two-third (66%, *n* = 347/526) who did not. Participants were also asked to indicate which social media sites they would prefer to use for seeking mental health support, 42% of the responses (*n* = 295/704) indicated none. YouTube was the most preferred site for seeking support with 19.7% of responses (*n* = 139/704) and WhatsApp thereafter with 12.5% of responses. Equal responses were provided for Facebook (6.8%) and Snapchat (6.7%). Instagram and Twitter were the least reported social media sites in this regard (Figure 3). The reasons behind using social media for support were further elicited by a contingency question. Interestingly, being less embarrassing was reported to be the main reason for using social media with 23.2% of responses (*n* = 102/439), followed by the ability to seek advice from people with similar problems (20.5%, *n* = 90/439), the ability to gain a wide range of opinions (17.3%, *n* = 76/439), and saving time (14.4%, *n* = 63/439). Participants also reported to seek social media support because they feel they will not be understood as highlighted in 13.9% of the responses (*n* = 61/439). Interestingly, privacy concerns was the least reported reason with 10.7% of responses (*n* = 47/439).

## Discussion

Using emoji facial expressions, more than half of the participating adolescents reported to be either neutral, sad, or very sad. In the current study, the bivariate analysis showed multiple factors to be associated with negative mental health, which is reflective of the literature. These include age, gender, smoking, drinking, discrimination, bullying, presence of health conditions, low physical activity, and high ST.

However, upon conducting multivariate analysis, it became clear that discrimination was the most predictive factor for negative mental health, with a fivefold increase in odds of having negative mental health feelings (a score of > 50%) among the participating adolescents. A plausible explanation of the current finding is related to the absence of well-established schemes for tackling discrimination at schools/colleges at the time of conducting the study. The detrimental effect of discrimination on both mental and physical health has been identified by several scholars in the literature whether among adults or adolescent (13–18, 36). Discrimination was experienced by 60% of the study sample, and 833 responses were provided regarding the causing factors, suggesting that the surveyed adolescents suffered from multiple forms of discrimination. This is more worrying given the results of a previous research which highlighted that experiencing multiple forms of discrimination is associated with worse mental and physical health above the effect of only one form (16).

Increased age was another risk factor affecting adolescents' mental health in the current study, similar to existing research. Unsurprisingly, a previous research by Kessler et al. (37) suggested that 50% of mental health conditions are established when individuals reach the age of fourteen. This percentage increases to 75% by the time the individuals become 24 years of age. Interestingly, another research reported increased odds of feeling depressed/stressed for each year of age among high school-aged adolescents (38). Given that, this suggests that the current study cohort is at high risk of developing further mental health disorders in the next few years. Additionally, this stresses the importance of early detection and tackling of mental health symptoms among adolescents. Female gender was also found to play a significant role in mental health status. This can be due to the fact that females go through mood and behavior fluctuation which occur in conjunction with the phases of menstrual cycle (39). In the current research, females were two times more likely to have negative mental feelings, which is consistent with previous findings in the literature (12, 38). In England, a previous research identified gender to be a significant predictor of depressive symptoms among students aged 11–14 years (12), with girls being twice as likely to show depressive symptoms as boys (12). Another study also showed that female gender had >3-fold-increased odds of reporting depression and stress (38).

Smoking was another risk factor identified to be significantly influencing mental health. In fact, there has been substantial evidence in the literature linking smoking to mental health disorders in adolescents (28–30). However, other scholars argue for this association to be more complex and bidirectional in nature (40, 41). There are many reasons for adolescents to engage in health-compromising behaviors such as smoking; these include childhood abuse, stressful life events, and depressive symptoms (42). Adolescent mental health can be a risk factor for adolescent smoking onset (41), given that adolescents can refer to smoking as a mean of self-medication to cope with psychological distress and depression (43, 44), thus building these social habits as an escape mechanism. Some studies have also demonstrated that alcohol and tobacco use during the last phase of adolescence can increase the risk of depression in adulthood (30). Given this context, it can be argued for a vicious cycle of association to be existing between social habits such as smoking and adolescents' mental health. This in return suggests that healthcare professionals should take into consideration the physiological and psychological consequences of smoking and drinking among adolescents when promoting mental well-being among adolescents.

The most commonly sought sources of support in order of frequency were friends, parents, searching the internet, and schoolteachers. Interestingly, family instability was found to be significantly associated with dominance of negative mental health feelings/symptomatologies among the study cohort. This could provide a plausible explanation as to why a third of the participating adolescents chose parents as the second source of support when having mental health problems. The current results also resonate with a previous research in Canada, which emphasized the importance for adolescents to have good relationships with family, friends, and healthcare providers in order to help them achieve control over their mental health condition (45). On the other hand, what was evident from the current study is that the use of social media was not considered as a main source of support. Interestingly, a previous qualitative research highlighted that adolescents perceived social media as a threat to mental well-being in terms of being a cause of mood and anxiety disorders, platform for cyberbullying, and a potential source of addiction (46), which in return could provide a potential explanation to the current results. Given these findings, it can be argued for the need to curb mental health issues on multiple levels including individual, community, and policymakers. As such, identification of sources of support becomes crucial in order to identify the best measures to tackle such a problem. For example, in response to Public Health England (PHE), health link workers are currently introduced into schools in England with the aim of delivering targeted interventions to adolescents including emotional health and well-being, smoking, and drug, alcohol, and substance misuse, to list a few. Additionally, link workers are also responsible for delivering training, support, and advice to school staff, young people, and parents about personal, social, and health education (PSHE) and health promotion. Adolescents spend most of their time with teachers, parents, and friends. Hence, schoolteachers, friends, and parents are best placed to recognize and support young people suffering with mental health issues.

### Study Limitations

The use of a non-validated surrogate measure rather than a validated tool to assess mental health issues constituted a major limitation in the current study. In addition, the study had other limitations, including exclusion of adolescents aged 10–11 years, exclusion of school/college dropouts, and non-school going adolescents. The study being observational in nature (non-randomized), using convenience sampling for recruitment, and being conducted in certain boroughs in Greater London, further limits the generalizability of the results on a national scale. Furthermore, studies employing self-administered questionnaires have other potential limitations including recall bias, non-response bias, and social desirability bias (47). Despite these limitations, the current study provides an important insight into the status quo of adolescents' mental health and predicting factors, thus contributing to the existing literature.

However, a study on a national scale with the use of a validated tool is highly recommended as its outcomes may provide an insight into the real magnitude of the problem across the nation.

## Conclusion

Several risk factors such as age, gender, social habits (smoking and alcohol consumption), discrimination, bullying, health comorbidities, and sedentary lifestyle were found to be associated with dominance of negative mental health feelings. Among these factors, discrimination was found to be the most predictive factor influencing negative symptomatology among the study cohort. Considering the wide spread of discrimination as unraveled by the current COVID-19 pandemic, a similar study on a national scale with the use of a validated tool is highly recommended to determine the real magnitude of the problem and its impact, which would be the gate for the proper tackling of mental health issues and associated complexities among the adolescent population across the nation.

## Data Availability Statement

The raw data supporting the conclusions of this article will be made available by the authors, without undue reservation.

## Ethics Statement

The studies involving human participants were reviewed and approved by the Science, Engineering and Computing Delegated Ethics Research Committee at Kingston University London (Ref: 1213/045). Written informed consent from the participants' legal guardian/next of kin was not required to participate in this study in accordance with the national legislation and the institutional requirements.

## Author Contributions

RK conceived of the study, participated in its design and coordination and contributed toward the critical revision on all versions of the manuscript. IH drafted the manuscript and contributed to literature search, data analysis, data management, and checking of the results. AA-Z contributed to study design, literature search, data collection, and data analysis. All authors read and approved the final manuscript.

## Conflict of Interest

The authors declare that the research was conducted in the absence of any commercial or financial relationships that could be construed as a potential conflict of interest.
