# Supplementary material for: Mental Health Among School-Going Adolescents in Greater London: A Cross-Sectional Study
Source: Front Psychiatry. 2021 Mar 19;12:592624. doi: 10.3389/fpsyt.2021.592624 (PMC8017155; doi:10.3389/fpsyt.2021.592624)
Supplement: Supplementary file 1 [file Data_Sheet_1.docx]

Appendix A: The study questionnaire

Let us know about your feelings and mental health

Let us know about your feelings is an anonymous questionnaire. Your responses will not be shared with anyone.

Thank you for participating, we just want to know how you feel and know more about your attitude about your mental health. This is part of my final year project and your help is very much needed. I wish you all the best.

**Section one: demographics**

1. What is your gender?


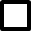
 Male


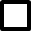
 Female


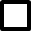
 Prefer not to state

1. What is your age category?


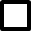
 11-12 years old


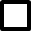
 12-13 years old


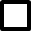
 13-14 years old


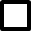
 14-15 years old


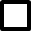
 15-16 years old


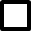
 16-17 years old


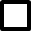
 17-18 years old


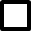
 18-19 years old

1. Were you born in the UK?
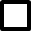
 Yes


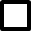
 No

If No, then how long have you been living here?

1. What is your ethnicity?


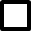
 White
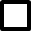
 Indian


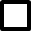
 Pakistani


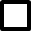
 Bangladeshi
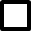
 African Black


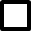
 Caribbean Black
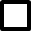
 Other Black


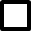
 Chinese


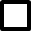
 Other Asian
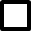
 Persian


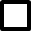
 Arab


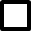
 Prefer not to state

1. What year are you in at the moment?
2. In which area is your school located?
3. Do you have any of the following? (Please tick all applicable)
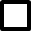
 Physical disability


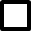
 Asthma
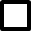
 Diabetes
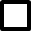
 ADHD


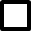
 Dyslexia
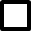
 Eczema
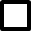
 Epilepsy


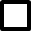
 Autism/ASD
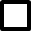
 Painful joints


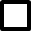
 Learning difficulties
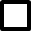
 None


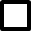
 Other, please state

**Section two: Lifestyle**

1. Do you smoke?


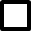
 No


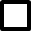
 Yes

If yes, then is that casual or regularly?

1. Do you drink alcohol?


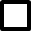
 No
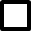
 Yes

If yes, then is that on regular basis or in parties only?

1. Apart from PE sessions, how often do you play sport a week?
2. What is your weekly income?
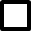
 None


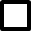
 Up to £5
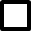
 £5-£10


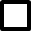
 More than £10

1. What do you usually prefer to have for lunch? (Please tick all applicable)
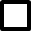
 Pizza


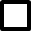
 Pasta/bread/ potatoes


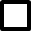

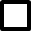

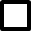
 Vegetables Meat/fish/egg Sandwiches

1. How many hours do you spend on your homework daily?
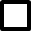
 None


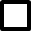
 Up to 1 hour
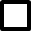
 1-2 hours


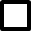
 2-3 hours


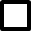
 More than 3 hours

1. Who do you live with?


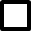
 Both parents


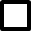
 Both parents and siblings
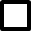
 Mother and siblings


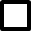
 Father and siblings


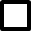
 Father or mother alone


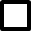
 Grandparents/ other family members
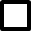
 Foster parents


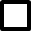
 I am in care

**Section three: Mental health evaluation**

1. How do you feel in general? (please circle one face only)


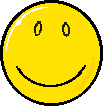

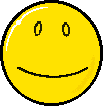

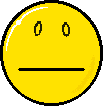

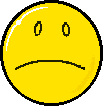

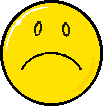
1 2 3 4 5

1. Have you experienced discrimination on the basis of any of the following? (Please tick all applicable)


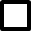
 Accent
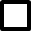
 Age

-
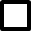
Disability
- Ethnicity

Language


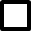
Nationality


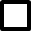
 Sex/gender


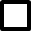
 Sexual orientation
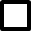
 Skin colour


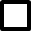
 Dress/appearance
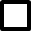
 Body size


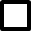
 None

- Other, please state

1. Who have you been discriminated by?


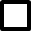

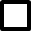

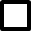

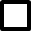

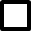
 Student
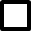
 Friend Teacher Public Police None


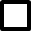
Other

1. Have you been bullied in the past 6 months?
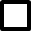
 No


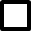
 Yes

If yes, then state where?


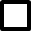

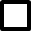
 School
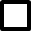
 Home Other

1. In the past 6 months, have you experienced any of the following symptoms? (Please tick all applicable)


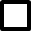
 I felt frequently sad, like I couldn’t go to school


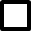
 I stopped having fun doing things that I used to enjoy
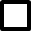
 I felt slowed down compared to my usual pace


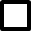
 I was frequently feeling up and down


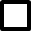
 I lost or gained weight without trying to, or my appetite have changed
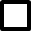
 I have been feeling good/ok all the time

1. Do you know any of your friends who are low in mood/ going through difficult times?
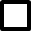
 No


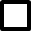
 Yes

If yes, then how do you support them?

1. Life can be challenging and may include stressful situations, which one of the following in your opinion affects you the most? (Please tick all applicable)

- Relationships difficulties
- Hectic schedules


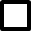
 Sudden change


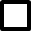
 Serious challenges Financial hardship Discrimination

Feelings of isolation

Unsafe neighbourhood

1. How often did you experience panic attacks in the last 6 months? Several times a day

Once or twice a day A few times a week

Less than few times a week Never

1. How frequently did you feel low in mood in the past 6 months? Several times a day

Once or twice a day A few times a week

Less than few times a week Never

1. In the past 6 months did you experience any of the following? (Please tick all applicable)

I was feeling anxious, worried or scarred about a lot of things in my life I felt that my worry was out of control

I felt restless, agitated and tense I had troubles sleeping

I could not fall or stay asleep

1. In the past year did you experience any of the following symptoms for long periods of time? (Please tick all applicable)

I felt exhausted

I felt worthless or guilty

I keep thinking about death I felt good

I felt great

**Section four: Use of social media**

1. Roughly how much time do you spend on your phone/laptop a day?
2. In general, which of the following social media do you use the most? (Please tick all applicable) Facebook

YouTube

Twitter

Snapchat WhatsApp BBM

Instagram None

Others, please specify

1. Who do you prefer to approach if you need to seek help or if you have any troubles with your mental health? (Please tick all applicable)

Parents Friends

- Health care professional
- GP

Internet Book

School teacher

Mobile application, please specify Social media site

Web page, please specify No one

1. Do you share your problems on social media sites to get support?

Yes

No

1. Do you refer to any of the following social media applications if you need help or support with your mental health? (Please tick all applicable)

Facebook

YouTube

Twitter

Snapchat

WhatsApp

Instagram

None

Others, please specify

1. If you use social media/mobile applications or specific websites for support, please indicate the reason? (Tick all applicable)

Privacy concerns

Don’t feel will be understood

Less embarrassing

Can seek advice from people with similar problems

Ability to gain wide range of opinions

Saves time

I do not use social media/ mobile applications

Others, please specify

*Thank you for your participation*
